# Ascariasis in a 75-year-old man with small bowel volvulus: a case report

**DOI:** 10.1186/s12879-021-06718-z

**Published:** 2021-10-09

**Authors:** Giovanni Romano, Paola Pepe, Serena Cavallero, Paola Cociancic, Lorenzo Di Libero, Giovanni Grande, Giuseppe Cringoli, Stefano D’Amelio, Laura Rinaldi

**Affiliations:** 1 Department of General Surgery, Frieri-Criscuoli Hospital, Sant’Angelo dei Lombardi, AV Italy; 2 Department of Veterinary Medicine and Animal Production, University of Naples Federico II, CREMOPAR, WHO Collaborating Centre for Diagnosis of Intestinal Helminths and Protozoa (ITA-116), Naples, Italy; 3 Department of Public Health and Infectious Diseases, Section of Parasitology, Sapienza University of Rome, Rome, Italy; 4 Centro de Estudios Parasitológicos y de Vectores (CEPAVE-CONICET-UNLP-asociado a CICPBA), La Plata, Buenos Aires Argentina

**Keywords:** Ascariasis, *Ascaris suum*, Human, Zoonosis, Pigs, Volvulus, Case report

## Abstract

**Background:**

*Ascaris lumbricoides* and *Ascaris suum* are the most common soil-transmitted helminths of humans and pigs, respectively. The zoonotic potential of *A. suum* has been a matter of debate for decades. This study was aimed to present a case of human ascariasis caused by *A. suum* in southern Italy.

**Case presentation:**

A 75-year-old man presented to the department of surgery in Avellino (southern Italy) complaining of abdominal pain and vomiting. Physical examination revealed bloating and abdominal tenderness. A computed tomography scan showed air-fluid levels and small bowel distension. During exploratory laparotomy a small bowel volvulus with mesenteritis was evident and surprisingly an intraluminal worm was detected. The worm was removed with a small enterotomy and identified as an adult female of *A. suum* based on morphological and molecular analysis. Faecal examination revealed the presence of unfertilized *Ascaris* eggs with an intensity of 16 eggs per gram (EPG) of faeces. The patient was treated with mebendanzole 100 mg twice a day for 3 days. The post-operative course was regular with re-alimentation after 3 days and discharge after 12 days.

**Conclusions:**

This report shows as *A. suum* can function as a relevant agent of human zoonosis. Therefore, in patients with bowel obstruction with no evident aetiology a helminthic infestation should be considered for an accurate diagnosis, especially in patients living in rural areas.

## Background

*Ascaris lumbricoides* (Linnaeus, 1758) and *Ascaris suum* (Goeze, 1782) are the most common soil-transmitted helminths (STHs) of humans and pigs, respectively.

Human ascariasis, caused by *A. lumbricoides*, is one of the neglected tropical diseases (NTDs) of greatest public health and socio-economic importance. It was estimated around 447 million people were infected in 2017 and its global burden contributes over 860,000 Disability-Adjusted Life Years (DALYs) [1]. *Ascaris suum* is one of the most common parasites in pigs, leading to huge economic losses in the pig industry linked mainly to reduced growth, poor feed conversion efficiency and costs of control in farms [2].

*Ascaris lumbricoides* and *A. suum* produce cross-infections between pigs and humans being species potentially zoonotic [3]. The eggs of both species are morphologically indistinguishable whereas the adults differ only in the shape of the lips and teeth measure detectable by electron microscope [4]. Due to their morphological similarities, there is currently an active debate if *A. lumbricoides* and *A. suum* are the same species [5] or whether they are different species as indicated by genetic evidence [6].

Ascariasis is generally asymptomatic both in infected humans and in pigs, however, can cause intestinal obstruction and perforation peritonitis when high parasitic loads are present. Furthermore, as *Ascaris* larvae develop, different stage-specific antigens are observed and various tissues are invaded, therefore the effects of infection differ over the course of larval migration and development [7]. As regards *A. lumbricoides*, chronic infections may also be associated with growth failure and cognitive delay in children [8].

*Ascaris* infection is transmitted via faecal contamination of the environment with parasite eggs. Because of this, human ascariasis is prevalent in developing countries where poor access to adequate sources of water, sanitation and hygiene, favour the transmission human-to-human [3]. Nevertheless, cases have been reported in Denmark [9], the Netherlands [10], United Kingdom [11], Austria [12], Japan [13], the United States of America [14] with the zoonotic potential of pig-derived *Ascaris* as a plausible source of human infection in developed countries. Cavallero et al. [15] confirmed pig as a source of human infections in Italy, and the zoonotic transmission was also reported in north-western Italy by Dutto and Petrosillo [16] that found a positive case of a hybrid genotype of *A. suum/lumbricoides* in a pig farmer. Despite the findings, the zoonotic potential is underestimated in these countries [12] and prevention measures have been seldom applied simultaneously in both humans and pigs. The present study reports the case of a 75-year-old male from southern Italy infected with *A. suum* who developed an adult intestinal worm.

## Case presentation

**Fig. 1:**
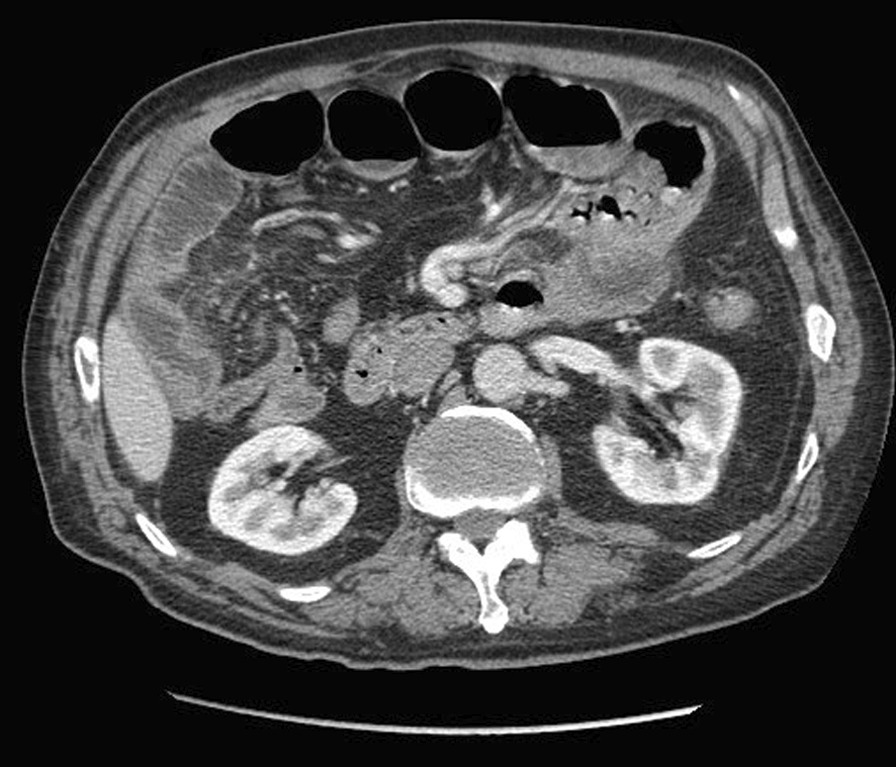
Computed tomography (CT) scan showed air-fluid levels and signs of mechanical ileus

**Fig. 2:**
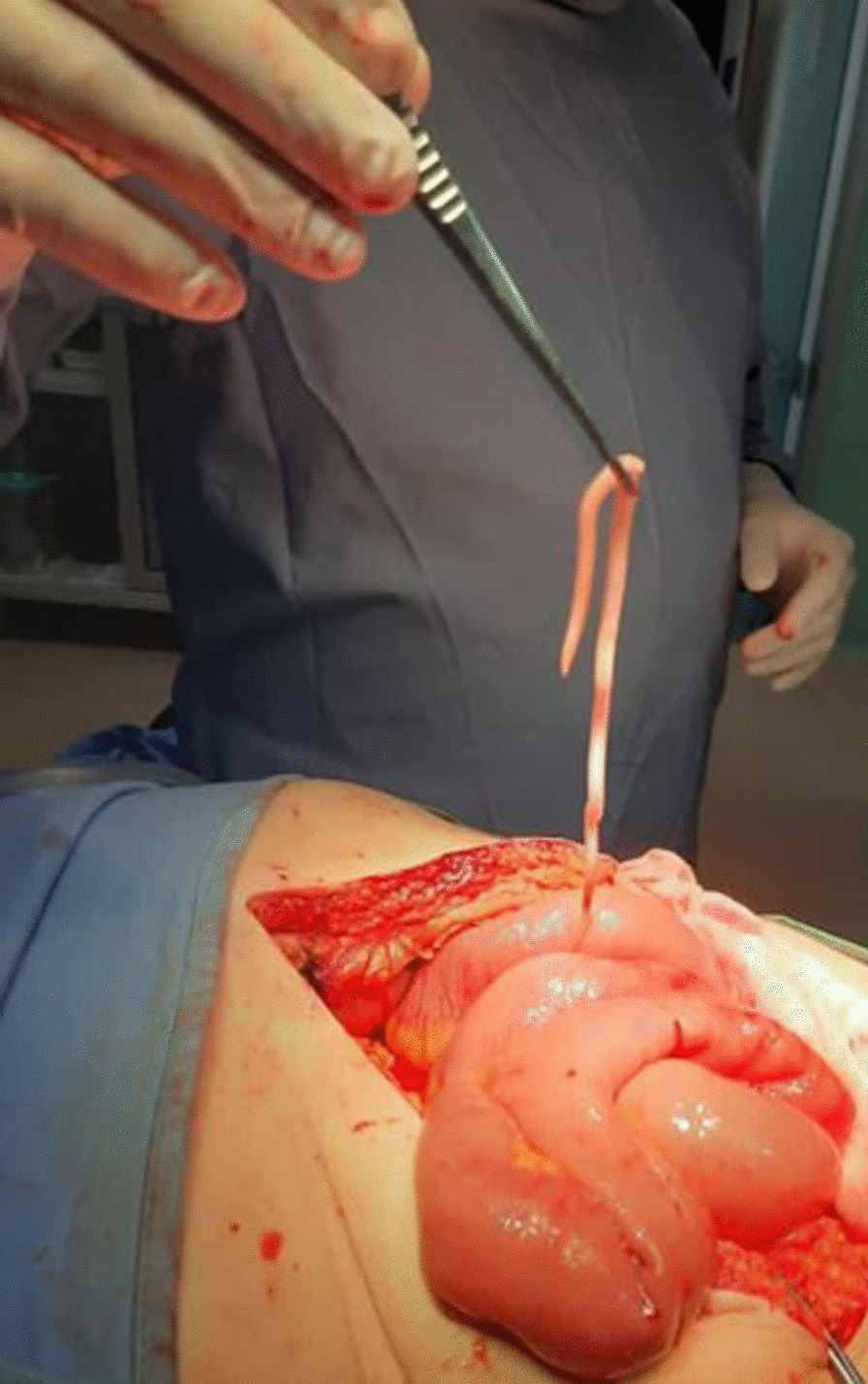
Extraction of the worm by sponge-holding forceps during the surgical intervention

In November 2020, a 75-year-old male with low comorbidity (hypertension, no previous abdominal surgery) was admitted to the Department of General Surgery, Frieri-Criscuoli Hospital (Avellino, Italy) with abdominal pain, vomiting and bowels not opened in the last 3 days. The patient lived in a rural area of the Campania region, southern Italy. The anamnesis revealed that until 3 years ago he had an orchard and raised chickens and pigs for his own consumption. Physical examination revealed abdominal pain with bloating. Examination of blood showed a white blood cell (WBC) count of 19.16 × 10^3^/μL with 86.8% neutrophils and 2.9% eosinophils. Computed tomography (CT) scan exposed multiple air-fluid levels in the epigastrium and left hypochondrium with some fluid in mesenteric recesses and signs of bowel obstruction (Fig. 1). The day after the hospitalization, the symptoms persisted so the patient was referred to surgery. The explorative laparotomy revealed bowel dilatation with vascular suffering and kinking of a tract of ileus in correspondence of a severe mesenteric inflammation. During the bowel palpation, from the Treitz ligament to the ileocecal valve, a long worm-like foreign body was felt and this occasioned to a little enterotomy to extract the worm (Fig. 2). The palpation of the entire bowel did not show the presence of other ones. The worm recovered was washed in saline solution and stored in 70% (v/v) ethanol. A stool sample was also collected from the bowel of the patient and preserved at + 4 °C.

The worm and the stool samples were sent, within 24 h, to the laboratories of the WHO Collaborating Centre for diagnosis of intestinal helminths and protozoa (WHO CCITA-116, University of Naples Federico II, Italy) for further analysis.

**Fig. 3:**
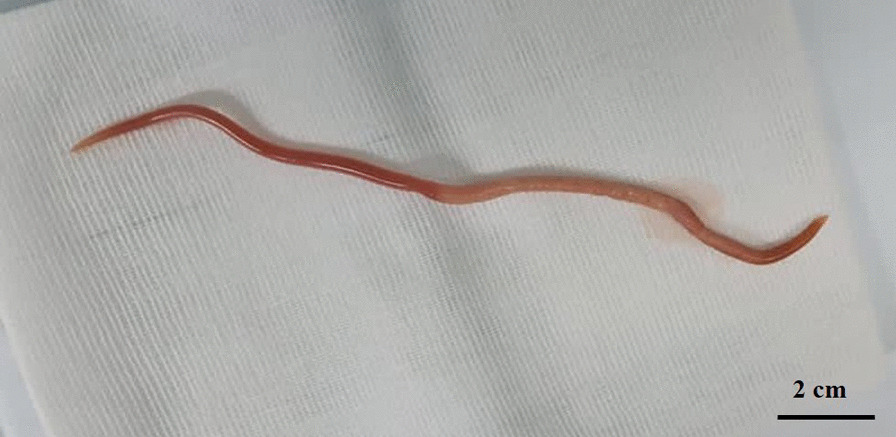
The adult worm recovered during the surgical intervention

**Fig. 4:**
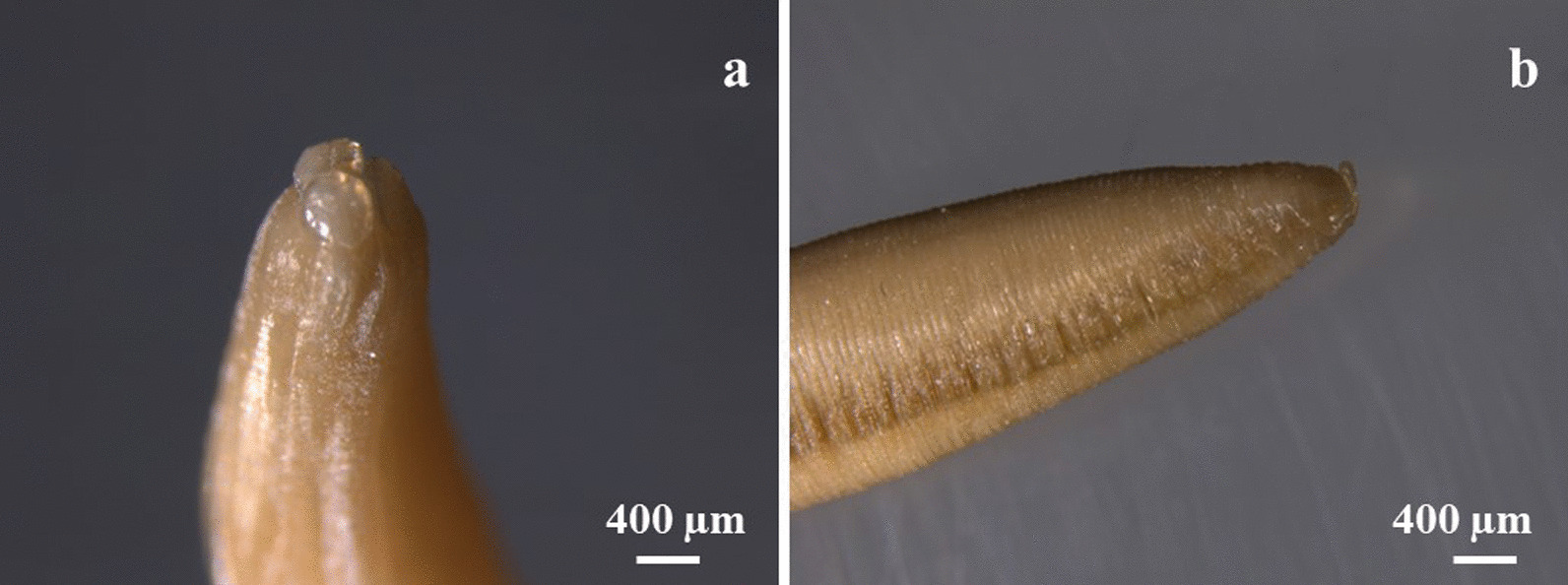
**a** Buccal orifice with three lips (two subventral and one dorsal); **b** Posterior end of the worm straight

The worm was cylindrical in shape, pinkish in colour and measured 20 cm in length and 6 mm in diameter (Fig. 3). Under the stereomicroscope the helminth presented a buccal orifice with three lips (two subventral and one dorsal) and a straight posterior end (Fig. 4). The worm was identified as an adult female of *Ascaris* spp. [17].

A portion of the adult worm was sent in 70% (v/v) ethanol to the Department of Public Health and Infectious Diseases, Section of Parasitology (Sapienza University of Rome, Italy) for the genetic characterization.

DNA extraction was performed using the Isolate II Genomic DNA kit (Bioline, UK), according to the manufacturer’s protocol. The entire ITS nuclear region (ITS1, 5.8S, ITS2) was amplified using 20 ng of genomic DNA, 10 mM Tris–HCl (pH 8.3), 1.5 mM MgCl2 (Bioline), 40 mM of a nucleotide mix (Bioline), 20 pmol/µl each of the forward primer NC5 (59-GTAGGTGAACCTGCGGAAGGATCAT- 39) and the reverse primer NC2 (59-TTAGTTTCTTCCTCCGCT-39) described by Zhu et al. [18] and 1.0 U of BIOTAQ DNA Polymerase (Bioline) in a final volume of 50 µl. The PCR was performed in a GenePro Eurocycler Dual Block (Bioer) under the following conditions: 10 min at 95 °C (initial denaturation), 30 cycles of 30 s at 95 °C (denaturation), 40 s at 52 °C (annealing) and 75 s at 72 °C (extension), and a final elongation step of 7 min at 72 °C. A negative control (PCR master mix without genomic DNA) was included in the amplification reaction.

**Fig. 5:**
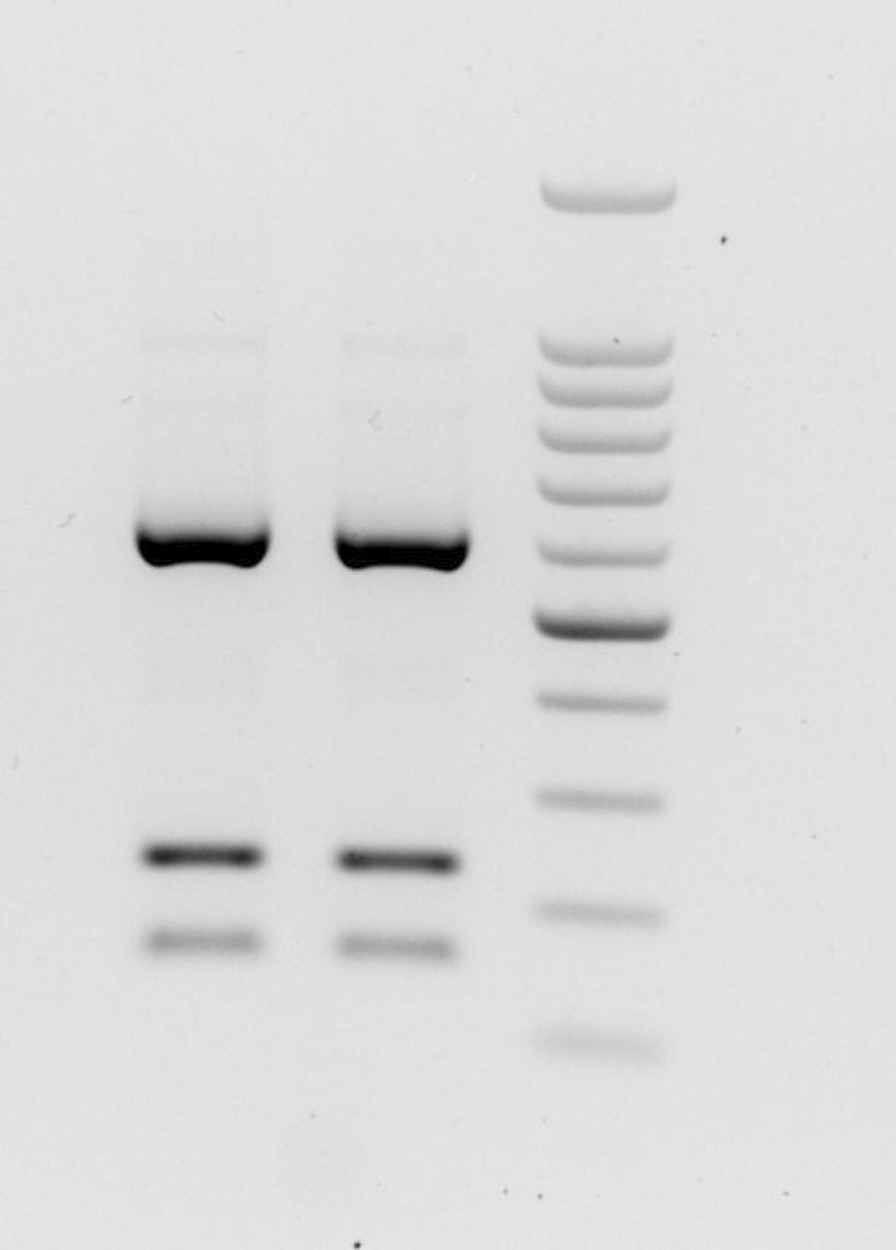
RFLP pattern obtained after digestion of complete ITS amplicon with *Hae*III along with a 100 bp ladder (highlighted band at 500 bp)

A PCR product of around 1000 bp was obtained. The ITS amplicon was digested with the restriction endonuclease *Hae*III, as the resulting patterns have been previously proved useful for the identification of *Ascaris* species. This approach yielded the specific banding pattern peculiar to the *A. suum* genotype, showing three bands of about 610 bp, 230 bp and 140 bp (Fig. 5).

**Fig. 6:**
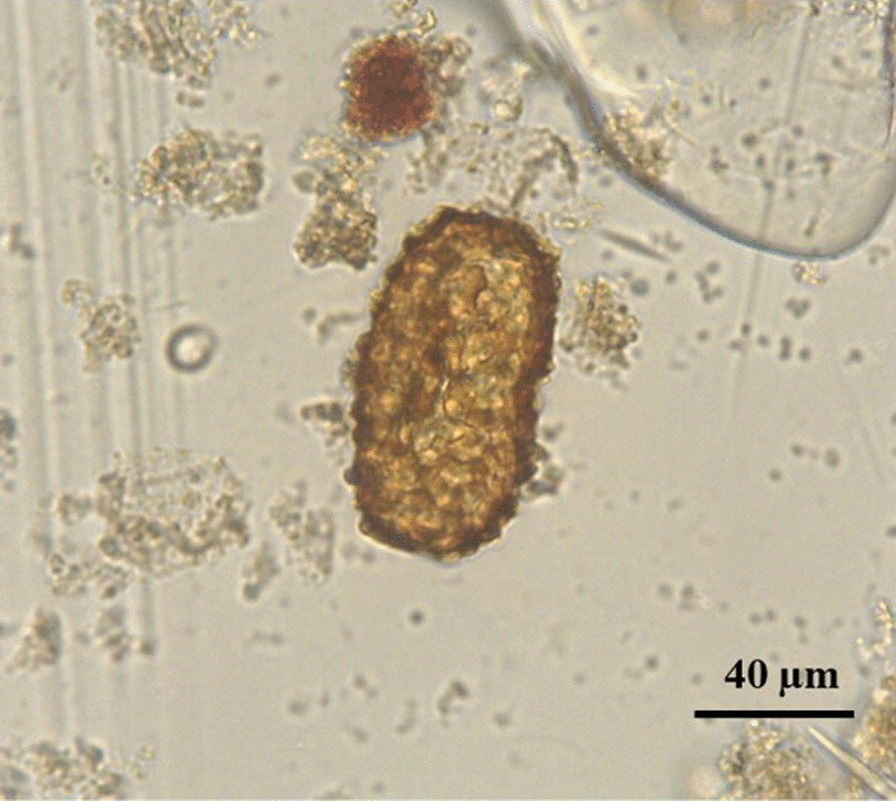
Unfertilized egg of *Ascaris* spp. detected by the FLOTAC technique

The stool sample was analysed with the FLOTAC dual technique [19] as described in Gualdieri et al. [20]. The analytic sensitivity was four eggs per gram (EPG) of faeces. Faecal examination revealed the presence of unfertilized *Ascaris* eggs (Fig. 6) with an intensity of 16 EPG.

The patient was treated with mebendazole 100 mg twice a day for 3 days. The stool examination at 7-days follow-up was negative. The post-operative hospitalization was regular with re-alimentation after 3 days and discharge after 12 days, asymptomatic. A week after the discharge another parasitological stool examination was negative.

## Discussion and conclusions

This report shows as *A. suum* can function as a relevant agent of human zoonosis in non-endemic areas. Human ascariasis is generally common in developing countries where access to personal hygiene and proper sanitation practices are not available; however, increased travel and migration have made this infection more common also in non-endemic areas [8]. Furthermore, sporadic cases of human ascariasis have been reported in developed countries in North America and Europe which have been mainly associated to *A. suum* [21], confirming that cross-transmission of *A. lumbricoides* and *A. suum* between humans and pigs likely occurred [15]. This is particularly likely in areas where humans and pigs live in close proximity, as demonstrated by Criscione et al. [22] that identified 4% and 7% of hybrids in adult samples of *Ascaris* collected in Guatemala and China, respectively. Therefore, under certain conditions in which people have frequent contact with pigs and soil, possible cross-infection with *Ascaris* should be considered for an accurate diagnosis.

*Ascaris suum* is widespread and high prevalence values have been reported in pigs in western countries like Denmark (25–88%) and Canada (18–82%) [23]. In Italy, *A. suum* infection is widely distributed in domestic pigs [24, 25]. In southern Italy, prevalence values of 20.1% and 88% have been reported in pigs (Cringoli et al., personal communication) and wild swine [26], respectively.

In the present study, *A. suum* was detected in a patient who had recently raised pigs in a rural area of southern Italy. Certain farm practices might have contributed to his exposure to *Ascaris* eggs, such as use of pig manure as fertilizer, use of pig bedding for compost, and location of pig pens near where produce is grown.

The definitive diagnosis of ascariasis was achieved only during the surgery through the bowel palpation from the Treitz ligament to the ileocecal valve because the clinical manifestations of the patient were very heterogeneous (e.g. abdominal pain, nausea, vomiting, etc.) and not specific for a helminth infection. Furthermore, it should be emphasized that bowel volvulus could be caused by other several factors such as intestinal malrotation (mostly in children), intestinal adhesions, decreased pelvic space due to pregnancy or pelvic mass, Hirschsprung or Chagas disease. For these reasons, diagnosis of infections by *Ascaris* with clinical symptoms, hematological investigations, and biochemical profile is usually inconclusive [27] due to the non-specific findings. Frequent symptoms reported are abdominal pain, nausea, vomiting, diarrhoea, and presence of worms in vomit or faeces [27–29]. During the physical examination, abdominal tenderness, bloating, abdominal mass, or rigidity could be presents whereas x-rays could reveal air fluid levels and shadow of roundworms in some cases [28]. Finally, laboratory findings associated to ascariasis are elevated leukocytosis, eosinophilia and elevated C-reactive protein [30]. An accurate diagnosis can be made by finding the eggs or adult worms in the stool. It is important to highlight that parasitological stool examination is not a routine test and that a low parasitic load cannot be detected if the technique used is not highly sensitive.

The finding of a single worm and the low parasitic intensity (16 EPG) diagnosed using the FLOTAC technique confirmed that zoonotic cases developed a smaller number of adult worms in the intestine when infected with eggs from pig *Ascaris* [9, 16], making the diagnosis even more difficult.

In this case report, the bowel obstruction was not caused by worm tangle but by a localized mesenteritis with bowel kinking. In patients with bowel obstruction and with a no evident aetiology a complete bowel palpation during surgery and a pre or post-operative parasitological examination should be performed.

Because of the economic and health importance of *Ascaris* infection, an integrated One Health approach based on farm practices, animal husbandry and health education efforts should be addressed to reduce its transmission [14].

Prevention measures in farms should include the use of equipment for handling animal waste and stall cleaning, a proper personal hygiene (wash hands before and after contact with pigs, pig waste, or soil contaminated with pig waste) and keeping pig pens separate from vegetable fields and avoiding use of pig manure as fertilizer [14]. Furthermore, since ascariasis in pigs is frequently sub-clinical, diagnostic techniques should be used routinely (e.g. ELISA, *post-mortem* examination of liver and lung lesions, faecal egg count) to estimate parasite presence in farms [31, 32]. Finally, because *Ascaris* eggs can remain viable for extended periods in soil, it should be recommended to wash thoroughly raw produce before consumption. Furthermore, preventive measures should be included in health education programmes to reduce the risk of infection.

## Data Availability

The datasets used and/or analysed during the current study available from the corresponding author on reasonable request.

## Abbreviations

STHs: Soil-transmitted helminths
NTDs: Neglected tropical diseases
DALYs: Disability-Adjusted Life Years
WBC: White blood cell
CT: Computed tomography
EPG: Eggs per gram
